# The association between patient engagement and treatment outcome in guided internet-delivered CBT for anxiety and depression

**DOI:** 10.3389/fpsyg.2025.1494729

**Published:** 2025-06-09

**Authors:** Karin Hammerfald, Henrik Haaland Jahren, Ole André Solbakken

**Affiliations:** ^1^Department of Psychology, University of Oslo, Oslo, Norway; ^2^Braive AS, Oslo, Norway

**Keywords:** anxiety, depression, iCBT, symptom change, predictors of change, user engagement, routine care

## Abstract

**Introduction:**

The present evaluation aimed to explore patterns in routinely collected clinical data to better understand how user engagement may be associated with symptom change during guided iCBT treatment for depression and anxiety in a routine care setting. As part of ongoing quality assurance efforts, we examined whether specific engagement indicators were related to treatment outcomes. These analyses were motivated by previous findings in the literature suggesting that higher engagement may be linked to greater symptom improvement.

**Methods:**

Anonymous data of 514 patients who signed up for an internet-delivered, guided treatment program for depression or anxiety, were obtained for estimating patterns of change and the impact of predictors of change using Multilevel Modeling. Initial assessment after sign-up included various questionnaires and demographic information. Log data from user interactions with the guided iCBT programs was used to assess patient and clinician engagement. Clinical outcomes included symptoms of depression (Patient Health Questionnaire, PHQ-9) and anxiety (Generalized Anxiety Disorder-7, GAD-7).

**Results:**

Patients started a mean of 7.14 modules, completed 64.7% of assigned modules and 62.8% of assigned activities. Patients with clinical depression or anxiety levels experienced significant changes between initial assessment and first outcome assessment as well as significant symptom reduction during treatment. Initial symptom levels and engagement persistence predicted treatment outcomes.

**Conclusions:**

The present study replicates previous findings suggesting that safeguarding exposure to and engagement with content is significantly associated with outcome.

## 1 Introduction

Depression and anxiety are the most common mental disorders (CMDs) worldwide (Bullis et al., 2019), with estimated lifetime prevalence rates of 9.7 and 12.9% respectively (Steel et al., 2014). CMDs make a significant contribution to the overall global disease burden (Whiteford et al., 2013), with devastating individual, societal, and economic effects (The Lancet Global Health, 2020). Despite numerous studies consistently demonstrating stable numbers, the global deterioration of mental health status throughout the COVID-19 pandemic has been observed (WHO, 2022). Simultaneously, the pandemic has spurred the adoption of technology in the realm of psychological interventions and treatment, rendering digital psychotherapy services more accessible, reducing waiting lists and costs (Rollman, 2018; Pfender, 2020).

Among the various internet-delivered treatments, the majority of research has focused on internet-based cognitive behavioral therapy (iCBT), supporting its effectiveness for anxiety and depression (Etzelmueller et al., 2020; Nordh et al., 2021; Rosenström et al., 2025), with a symptom reduction comparable to face-to-face CBT (Carlbring et al., 2018; Andersson et al., 2019; Andrews et al., 2018). Many studies on internet-delivered interventions have primarily focused on analyzing outcomes at fixed time points and have often overlooked how the program is adopted and used by the users (Sieverink et al., 2017). To ensure that digital mental health interventions can make a meaningful impact, individuals must receive a “therapeutic dose." This necessitates continued engagement with effective interventions. Engagement metrics have long focused on dropout attrition in terms of the rate of participants who do not complete the per-protocol treatment (Eysenbach, 2005). However, compliance as a function of the user interaction with the platform might allow for deeper insights into the dose-response relationship of internet-delivered interventions (Donkin et al., 2013; Christensen et al., 2009; van Ballegooijen et al., 2014). As usage aspects can be easily captured via automatically collected log data, a broader range of adherence characteristics could be used, providing comprehensive and profound insights into user engagement throughout a treatment process (van Gemert-Pijnen et al., 2014). The crucial question, however, is which usage aspects are relevant for outcomes. Usage of internet-delivered interventions has previously been categorized into distinct categories, such as active vs. passive engagement (Enrique et al., 2019) and depth and breadth of engagement (Couper et al., 2010), to differentiate between users who actively interact with the program and complete the assigned tasks from those who only superficially go through the program and demonstrate limited interaction with it.

Overall, the existing body of literature on the topic suggests that a positive relationship between platform usage and treatment outcomes exists, both for clinical trials (Couper et al., 2010; Donkin et al., 2013; Enrique et al., 2019; Fuhr et al., 2018) and routine care (Staples et al., 2019). Yet, a review examining the influence of adherence on the efficacy of digital interventions discovered that usage metrics did not consistently correlate with improvements in symptoms (Donkin et al., 2011). Completing a higher proportion of modules, though, was invariably associated with positive outcomes (Donkin et al., 2011).

The present study set out to investigate the relationship between user engagement on symptom development while completing guided iCBT treatment aimed at reducing symptoms of depression and/or anxiety in a routine care setting. Based on the existing literature, we predicted that usage would be positively associated with treatment outcomes. Specifically, we expected that modules completed would have the strongest association with outcomes.

## 2 Materials and methods

### 2.1 Study setting and participants

The present study used routinely collected clinical measures and user behavior data from Braive. Braive is a low-threshold, scalable, digital on-demand solution offering psychotherapy treatment programs for people suffering from CMD. Braive collaborates with insurance companies, hospitals, and private practitioners to deliver either self-help or guided treatment. After being handed out a written informed consent form that they can agree to or reject, all patients undergo an initial diagnostic assessment. This assessment is based on the so-called Mental Health Check (MHC; thoroughly presented under 2.3.1.1) and a subsequent 30-min video call with a clinician. Based on the MHC results and the clinical evaluation, one of 12 treatment programs is recommended to the patient. During treatment, patients complete self-report questionnaires, for as long as they remain active in the program. These measures are collected at the beginning of treatment and repeatedly during treatment to track symptom development, and are a compulsory treatment element. Which of these questionnaires the patients complete and in which module they complete them depends on which treatment program they enrolled in.

#### 2.1.1 Online intervention

All programs were delivered on an online platform in a blended treatment format. Treatment programs consist of 10 to 12 treatment modules, each designed to be completed within 1 week. The programs are structured sequentially, meaning that earlier lessons must be completed before participants can access later ones. Braive differentiates between mandatory and optional activities, and all platform-defined mandatory activities need to be completed before moving on to the next module. Each module follows a structured format, containing well-proven CBT techniques delivered via animated psychoeducational videos, interactive activities, audio exercises, and a comprehensive toolbox. Clinicians give feedback on completed modules in asynchronous written form via an integrated chat function or in synchronous video calls, varying depending on the patient's needs. In this patient sample, analyses were restricted to patients completing one of four treatment programs aimed at targeting symptoms of depression and/or anxiety: “Depression and Sadness," “Depression and Social Anxiety," “Mixed Depression and Anxiety," and “Worry and Anxiety." Therapies were conducted by Braive psychologists in Norway and Sweden and by clinicians working at one of Braive's collaborating institutions. Participants received the program as part of a prescribed mental health treatment.

#### 2.1.2 Therapists

Patients were allocated to one of 42 clinicians working at Braive or one of Braive's partner institutions. All clinicians were nationally registered psychologists. Therapist guidance throughout treatment consisted of weekly feedback, either in the chat function or a 20-min video call. As clinicians had access to the patient platform and see the patient's activities and progress, the written feedback was individually tailored to the patient's challenges.

#### 2.1.3 Original dataset

The original dataset comprised 918 patients signing up for a Braive program between September 22, 2021, and April 20, 2023, completing at least 2 treatment modules and having signed the informed consent form (33 of 951 patients did not consent to the use of their anonymized data). The data routinely collected by Braive includes demographic information, outcome measures, and information about treatment modalities in terms of assigned clinician, selected treatment program, and number of modules completed. Exclusion criteria for Braive treatment are active suicidality or self-harm, ongoing drug abuse or hazardous substance usage, major reading and writing difficulties, major language barriers, or a diagnosis of mental health disorder with psychotic features.

#### 2.1.4 Study-specific dataset

We removed 394 individuals who received other treatment programs at Braive (i.e., youth programs, couples therapy, stress prevention programs). We considered MHC assessments to be invalid if they were collected more than 4 weeks before the beginning of the first iCBT module. Based on this definition, we excluded 10 more participants from the study. We restricted our analyses to the remaining 514 patients who completed at least two treatment modules within one of four treatment programs aimed at treating depression or anxiety. However, sample sizes varied across analyses and are specifically addressed in the respective sections. The patients' age is assessed by an age range (16–19: *n* = 5; 20–25: *n* = 62; 26–34: *n* = 230; 35-44: *n* = 123; 45–65: *n* = 91; 65+: *n* = 3), therefore a mean could not be computed. The modal age of patients in the study sample was 26–34 years, and 53% of the patients were female (*n* = 271).

### 2.2 Ethics statement

Only patients signing an informed consent for their anonymous data to be used in routine evaluations for service monitoring and improvement were included in the data analysis. As the data analysis presented in this paper falls under the umbrella of quality assurance and therefore outside the scope of the Health Research Act, no ethical approval was needed from the Regional Committee for Medical and Health Research Ethics in Norway (REK). The data protection officer at Braive approved the sharing of the data per a data protection agreement between Braive and UiO. All data analyses were done in compliance with the principles of the Declaration of Helsinki (WMA, 2013).

### 2.3 Measures

#### 2.3.1 Intake measures

Before starting a treatment program, all patients undergo initial diagnostic assessment.

##### 2.3.1.1 The Mental Health Check (MHC)

The MHC comprises demographic variables and a set of validated psychometric questionnaires to assess the patient's mental health condition, including the Insomnia Severity Index ISI (Chalder, 1996), the Patient Health Questionnaire PHQ, 4-item and 9-item version (Kroenke and Spitzer, 2002; Kroenke et al., 2009), the Karolinska Exhaustion Disorder Scale KEDS (Besèr et al., 2014), the Generalized Anxiety Disorder 7-item scale GAD-7(Spitzer et al., 2006), the Social Phobia Inventory short version Mini-SPIN (Connor et al., 2001), the Perceived Stress Scale PSS, 4-item and 9-item version (Cohen et al., 1983), the Panic Disorder Screener PADIS (Batterham et al., 2015), the Global assessment of functioning GAF (Hall, 1995), the Iowa Personality Disorder Screen IPDS (Langbehn et al., 1999), the Primary Care PTSD Screen for DSM-5 (PC-PTSD-5) (Prins et al., 2016), the Brief Grief Questionnaire BGQ (Ito et al., 2012; Patel et al., 2019), the Body Dysmorphic Disorder Questionnaire BDDQ (Mancuso et al., 2010), the eating disorder screening instrument SCOFF (Morgan et al., 2000), the TAPS Tool (McNeely et al., 2016) and the Brief Biosocial Gambling Screen BBGS (Gebauer et al., 2010). The decision tree logic in the MHC uses screening questions. If they indicate the presence of symptoms in one area, more questions are released. This implies that MHC questions may vary based on the patient's specific mental health challenges. For instance, if a patient scores above the cut-off (≥2) on the anxiety-related items (items 1 and 2) of the PHQ-4, the GAD-7 is subsequently administered.

##### 2.3.1.2 Sociodemographic variables

Gender and age range were included in the statistical analyses as control variables.

#### 2.3.2 Outcome measures

Routine outcome monitoring (ROM) involved the use of the PHQ-9 for the depression program, the GAD-7 for the anxiety program, and both measures for the mixed depression and anxiety programs. Outcome measures were administered regularly, but in different modules depending on the treatment program.

##### 2.3.2.1 The Patient Health Questionnaire (PHQ-9)

The 9-item version of the PHQ (Kroenke and Spitzer, 2002) is a tool for measuring symptoms of depression according to the DSM-IV criteria for major depression. Scores for items are assigned on a scale ranging from 0 (*not at all*) to 3 (*nearly every day*), so the total score ranges between 0 and 27. Research has shown good psychometric properties and responsiveness to change in both in-person and online settings (Kroenke et al., 2010; Erbe et al., 2016; Titov et al., 2011; Löwe et al., 2004). The PHQ-9 has demonstrated acceptable to good internal consistency, with a Cronbach's alpha score ranging from 0.78 to 0.89 (Kroenke and Spitzer, 2002). The PHQ-9 is valid in both general and primary care populations (Martin et al., 2006; Cameron et al., 2008). The severity of depression is categorized as follows: *minimal* (0–4), *mild* (5–9), *moderate* (10–14), *moderately severe* (15–19), and *severe* (20–27). For this paper, we used a cut-off score of 10 (McMillan et al., 2010).

##### 2.3.2.2 The Generalized Anxiety Disorder 7-item scale (GAD-7)

The GAD-7 (Spitzer et al., 2006) is a tool for assessing generalized anxiety symptoms according to DSM-IV criteria. It includes seven questions rating on a 4-point Likert scale from 0 (*not at all*) to 3 (*nearly every day*) the intensity of a given symptom over the last 2 weeks. It has demonstrated strong psychometric properties and sensitivity to treatment-related change over time (Beard and Björgvinsson, 2014; Plummer et al., 2016). Studies have shown good to acceptable internal consistency for the GAD-7 (Cronbach's alpha ranging from 0.83 to 0.90) in internet-delivered treatment in various randomized controlled trials (Dear et al., 2016; Titov et al., 2013; Terides et al., 2018). The GAD-7 has four severity categories: *minimal* (0–4), *mild* (5–9), *moderate* (10–14), and *severe* (15–21). A score of 10 or greater on the GAD-7 has proven to be a reasonable cut-off point for identifying cases of generalized anxiety disorder (Spitzer et al., 2006).

### 2.4 Usage metrics

Several metrics were employed to evaluate the utilization of the treatment platform from MHC assessment to post-treatment.

#### 2.4.1 Patient log data

##### 2.4.1.1 Number of started modules

The four programs of interest consisted of either 10 or 12 modules. Braive records the number of started modules for each patient. Patients can start a new module as soon as they have completed all mandatory activities in the previous module. A module is considered started as soon as the first activity in this module is completed.

##### 2.4.1.2 Number of completed activities

Activities are spread out across modules, so users need to complete more modules in order to complete more activities and vice versa. The definition of activity completion depends on the type of activity, e.g., an allocated number of seconds for watching a psychoeducational video or a certain number of text boxes when doing a written homework task.

##### 2.4.1.3 Total number of logins

Number of times the user logs into the program throughout treatment. After 30 min of inactivity, the user is automatically logged out, and a new session is counted if the user logs in again.

##### 2.4.1.4 Total time in the program

The total time spent logged into the program in minutes. The system collects time stamps at the beginning and end of each page view. The time spent on the final page for each login was capped at 10 min, and the total time was calculated by adding up all page view durations.

##### 2.4.1.5 Total number of words

Total number of words a patient sends to their clinician in the chat function.

##### 2.4.1.6 Total number of messages sent

Total number of chat messages sent from patient to clinician.

#### 2.4.2 Clinician log data

##### 2.4.2.1 Total number of words

Total number of words sent from clinician to patient in the chat function.

##### 2.4.2.2 Total number of messages sent

Total number of messages sent from clinician to patient in the chat function.

#### 2.4.3 Factors of engagement

Due to multicollinearity between the user metrics variables (see Table 1), principal component analysis (PCA) for de-noising and data compression was applied to create more parsimonious summary measures of engagement. All grand mean-centered user metrics variables were subjected to a principal components analysis (PCA) in SPSS, version 29.0, for patients and clinicians separately. Four factors had an Eigenvalue above 1, with the Varimax rotated component matrix loading high on two variables each for each factor (see Table 2). Based on this, the following factors were extracted:

**Table 1:** User metrics correlation matrix.

**Intercorrelation of engagement factors**			
	**EI**	**WI**	**CE**
EP	−0.028^*^	0.008^*^	0.219^**^
EI	-	0.024	0.134^**^
WI	-	-	0.657^**^

EP, Engagement Persistence; EI, Engagement Intensity; WI, Written Interaction with Clinician; CE, clinician engagement; ^*^ *p* < 0.05; ^**^ *p* < 0.01.

**Table 2:** User metrics component matrix: principal component analysis with all grand mean-centered user metrics variables.

**User group**	**User metrics**	**Rotated components with eigenvalue** > **1**			
		**1**	**2**	**3**	**4**
Patient	Completed activities	**0.978**	0.165	0.108	-
	Number of started modules	**0.975**	0.173	0.119	-
	Total number of words	0.103	0.130	**0.899**	-
	Total number of messages	0.101	0.103	**0.904**	-
	Total number of logins	0.185	**0.935**	0.166	-
	Total session length in minutes	0.147	**0.954**	0.090	-
Clinician	Total number of words	-	-	-	**0.825**
	Total number of messages	-	-	-	**0.825**
	Explained variance in %	49.05	22.71	20.55	68.14

Extraction method: principal component analysis; Rotation method: Varimax with Kaiser normalization. Bold indicates variables loading high on the respective factors.

Factor 1: *Persistence of engagement* is a summary measure of how much content patients engaged in within the assigned program. The number of started modules and the number of completed activities loaded positively on the first principal component.

Factor 2: *Intensity of engagement* is a summary measure of how deeply patients engaged in the program, with the number of logins and the total number of minutes spent on the platform loading positively on the second principal component.

Factor 3: *Written interaction with clinician* is a summary measure of written communication with the assigned clinician from the patient's perspective. The total number of messages sent from patient to clinician and the total number of words in these messages load positively on this factor.

Factor 4: *Written clinician engagement* is a summary measure of how much the clinician engaged in writing with the patient, with the total number of messages sent and the total number of words in these messages loading positively on this factor. These components together accounted for 92.3% of the variance in the PCA and were included in the mixed linear models as predictor variables.

#### 2.4.4 Preparatory data analysis

For the outcome variables, the last available score was carried forward to the end of the time series and used as an additional measurement point. To assess potential bias resulting from the imputation process, analyses performed using both the imputed and non-imputed datasets were compared. The outcomes were practically identical.

When looking more closely at the data, we realized that a substantial number of patients started treatment below the cut-off in the outcome measure of interest. To investigate whether the effectiveness of the guided iCBT programs spans across different levels of symptom severity, we conducted separate outcome analyses for subclinical ( ≤ 9; GAD-7: *n* = 183, PHQ-9: *n* = 89) vs. clinical (≥10; GAD-7: *n* = 130, PHQ-9: *n* = 285) cases.

### 2.5 Statistical analyses

We employed multilevel modeling by utilizing the linear mixed models feature within SPSS, version 29.0. The statistical concepts and methodology of multilevel modeling (MLM) have been described in detail elsewhere (Singer and Willett, 2003; Snijders, 2012). MLM has demonstrated superior performance compared to conventional approaches in addressing missing data and mitigating potential dropout bias (Hamer and Simpson, 2009).

In the present study, we were interested in predicting response to treatment as a function of user engagement. Treatment start and termination were treated as fixed occasions and centered at zero, while assessments conducted during treatment were encoded to represent the relative timing of assessment through the measurement series for each course of therapy. This guaranteed that the measurements in the time series for each patient were arranged in a manner that preserves and acknowledges the relative time intervals between each measurement occasion.

To capture treatment effects before engaging in treatment modules, a paired-sample *t*-test was used to analyze changes in PHQ-9 and GAD-7 between MHC assessment at intake and the first point of measurement after starting treatment.^1^

To estimate variations in the magnitude of change on the two outcome variables for patients with either subclinical or clinical symptom levels, Cohen's *d* effect sizes were calculated for patients above and below the cut-off in the outcome variables for the initial assessment phase and the online intervention phase separately, thereby taking into account the mean number of sessions.

#### 2.5.1 Multilevel modeling

We computed standard linear multilevel models for the two outcome measures (PHQ-9 and GAD-7) separately, looking at the entire treatment trajectory vs. the module completion phase alone for subclinical and clinical patients. The multilevel models comprised two levels of analysis, with repeated measurements over time being nested within individuals. All predictors and control variables were time-invariant and thus included at level 2. Continuous predictor variables were grand mean-centered before the analyses. As there were high levels of variability in terms of patient load and a substantial number of therapists were responsible for treating just one patient, none of our models incorporated the nesting of treatments within therapists. To assess the variation in symptom severity across times of measurement, we started by computing a null model that included only the fixed effect of the centered time variable and a random effect of the intercept (Model 0). In the next step, we added a random effect of time (Model 1) to allow slopes to vary independently across patients.

Subsequently, we analyzed the impact of control and predictor variables on the model for PHQ-9 and GAD-7 separately. A dichotomous symptom level variable based on the previously defined cut-off scores for PHQ-9 and GAD-7 (subclinical vs. clinical) was entered (Model 2). Next, age and gender were included as control variables (Model 3). Then, user engagement factors (see Section 2.4) were entered (Model 4). If a particular predictor or control variable did not exhibit a significant impact on either the intercept or slope at the *p* ≤ 0.1 level, it was excluded in the subsequent step of the analysis. In the final step (Model 5), all significant predictors were retained.

To gauge the extent of the predictors' influence on changes in the general outcome variables, we computed a pseudo-*R*^2^ statistic (~*R*^2^) based on the formula of Bryk (1992). This metric signifies the proportion of variance in slopes that can be attributed to the incremental inclusion of control and predictor variables. To prevent the underestimation of error and the exaggeration of effect sizes, we normalized the estimated overall changes by dividing them by the combined standard deviations across all measurement points on the outcome variables. We adhered to Cohen's (1988) criteria for assessing effect sizes: small (*d* ≥ 0.2 and < 0.5), medium (*d* ≥ 0.5 and < 0.8), and large (*d* ≥ 0.8).

## 3 Results

The mean number of days between MHC completion and course start was 8.96 (SD = 15.11). On average, patients started 64.7% of their assigned modules (M = 7.08; SD = 3.67) and completed 62.8% of their assigned activities (M = 92.08; SD = 50.68). The mean number of logins was 34.24 (SD = 36.53) and the total session length in minutes was 637.88 (SD = 869.44). Patients, on average, wrote 14 messages to their clinician (M = 13.52; SD = 12.01) and received 19 messages from their clinician (M = 19.32; SD = 12.73). Details of the multilevel models estimating and predicting change in symptom severity are shown in Table 3. In each model, the intercept represents the estimated average starting point, time signifies the average estimated change from one module to the next, and the interaction terms signify the estimated impacts of the predictors on the overall change.

**Table 3:** Results of multilevel growth curve analysis: mean estimates of intercepts and rates of change in depression and anxiety symptoms for the module completion phase alone vs. the entire treatment trajectory for subclinical and clinical patients (with imputation).

		**PHQ-9**				**GAD-7**			
		**Subclinical (*****n*** = **89)**		**Clinical (*****n*** = **285)**		**Subclinical (*****n*** = **183)**		**Clinical (*****n*** = **130)**	
	**Models**	**Model 0**	**Model 1**	**Model 0**	**Model 1**	**Model 0**	**Model 1**	**Model 0**	**Model 1**
		**Est**	**Est**	**Est**	**Est**	**Est**	**Est**	**Est**	**Est**
	*Fixed effects*								
Module completion	Intercept	6.854 (0.264)	6.900 (0.198)	14.624 (0.286)^**^	14.615 (0.225)	6.107 (0.198)^**^	6.107 (0.164)	12.443 (0.379)^**^	12.431 (0.260)
	Time	−0.106 (0.021)^**^	−0.102 (0.024)^**^	−0.354 (0.021)^**^	−0.338 (0.025)^**^	−0.097 (0.017)^**^	−0.092 (0.020)^**^	−0.339 (0.030)^**^	−0.324 (0.049)^**^
	Residual	3.408 (0.302)^**^	2.880 (0.309)^**^	11.062 (0.527)^**^	8.941 (0.504)^**^	3.618 (0.206)^**^	2.918 (0.196)^**^	8.494 (0.579)^**^	7.311 (0.547)^**^
	Variance in intercept	4.010 (0.765)^**^	1.670 (0.573)^**^	16.403 (1.650)^**^	8.913 (1.229)^**^	4.470 (0.570)^**^	2.728 (0.196)^**^	12.381 (1.838)^**^	3.484 (1.178)^**^
	Variance in slopes	-	0.018 (0.008)^*^	-	0.077 (0.016)^**^	-	0.032 (0.008)^**^	-	-
	AIC	1553.09	1520	6670.91	6559.56	3647.53	3592.04	3061.53	3029.16
		Subclinical (*n* = 65)		Clinical (*n* = 261)		Subclinical (*n* = 141)		Clinical (*n* = 117)	
Sign-up to end of therapy	Intercept	7.907 (0.566)^**^	7.93 (−0.144)^**^	14.846 (0.261)^**^	14.869 (0.224)^**^	7.610 (.0471)^**^	7.636 (0.335)^**^	11.749 (0.247)^**^	11.77 (0.200)^**^
	Time	−0.154)^**^	−0.144 (0.054)^**^	−0.394 (0.018)^**^	−0.378 (0.023)^**^	−0.259 (0.042)^**^	−0.252 (0.056)^**^	−0.421 (.0.20)^**^	−0.413 (0.022)^**^
	Residual	4.138 (0.639)^**^	2.693 (0.476)^**^	11.597 (0.469)^**^	9.268 (0.428)^**^	3.903 (0.592)^**^	3.070 (0.525)^**^	10.148 (0.449)^**^	9.266 (0.465)^**^
	Variance in intercept	5.544 (1.975)^**^	1.934 (0.961)^*^	15.517 (1.484)^**^	11.233 (1.256)^**^	2.607 (1.164)^*^	0.805 (0.791)	9.709 (1.091)^**^	5.090 (0.895)^**^
	Variance in slopes	-	0.016 (0.018)	-	0.080 (0.013)^**^	-	0.035 (0.021)	-	0.034 (0.012)^**^
	AIC	510.59	483.19	8701.21	8584.46	498.77	488.72	6941.76	6879.21

Standard errors are in parenthesis. Estimations were done by the method of restricted maximum likelihood (REML). Model 0 on each outcome variable keeps rates of change constant across patients, while Model 1 allows rates of change to vary. As can be seen by the significant variance in slopes for the PHQ-9 and GAD-7 total distress and corresponding decrease in the AIC-fit index from Models 0 to 1, Model 1 is preferable in this case. ^*^p < 0.05; ^**^p < 0.01.

### 3.1 Depression symptoms

#### 3.1.1 Symptom change between MHC completion and first point of measurement

To analyze changes in PHQ-9 between MHC completion and the first point of measurement after starting treatment (module 1), we conducted a paired-sample *t*-test. The MHC logic implies that the PHQ-9 is only released if a patient scores above two in questions 1 and 2 of the PHQ-4. Thus, all patients completing a depression course despite having scored low on the PHQ-4 and therefore missing scores in the PHQ-9 at the initial assessment had to be excluded from analyses. In the remaining patient sample (*N* = 326), depressive symptoms decreased significantly between MHC completion and the first assessment during depression treatment (module 1) (*t-value* = 6.218, *df* = 325; *p* < 0.001). Across all patients, effect sizes in the assessment phase were small for the PHQ-9, with Cohen's *d* being estimated at 0.344.

#### 3.1.2 Symptom change during program completion

In the next step, we analyzed the change in PHQ-9 between the first and last treatment module after starting treatment with Mixed Linear Models, separated by symptom level, and carrying the last point of measurement forward. Results of the multilevel models for the PHQ-9 from the first to last assessment during treatment are presented in Table 3. Statistically significant improvements during treatment were found in depression scores for both subclinical (*n* = 89) and clinical (*n* = 285) symptom levels. When examining the outcome variables more closely, one sees that for the total score of the PHQ-9, the intercept (mean baseline value across the patients' individually calculated growth curves) was estimated at 6.90 for patients below cut-off at the beginning of treatment and 14.62 for patients above cut-off at the beginning of treatment. The estimated decrease per module was 0.10 points for subclinical patients and 0.38 points for clinical patients, yielding an overall estimated change across the treatment of -3.8 points for clinical patients when completing 10 modules (depression program, depression and anxiety program) and -4.56 points when completing 12 modules (depression and social anxiety program). For subclinical patients, the estimated symptom reduction assuming course completion was 1.0 points in the case of 10 modules and 1.2 points in the case of 12 modules. This corresponds to a small to medium effect size for clinical patients (Cohen's *d* = 0.426) and a negligibly small effect for subclinical patients (Cohen's *d* = 0.120). To reach a change in depressive symptoms ≥ RCI = 7.39 based on a non-clinical sample (Cameron et al., 2008), 19.6 modules would have been necessary for Braive patients starting treatment above cut-off.

#### 3.1.3 User metrics predictors of symptom change

Results of the multilevel models estimating and predicting change in PHQ-9 scores are shown in Table 4. We found a significant negative effect of the initial symptom level and the patient's engagement persistence on the developmental trajectory for the PHQ-9. This means that patients with higher overall symptom scores at the beginning of treatment and with more started modules and completed activities demonstrate greater changes in depression levels during the online intervention. Differences in symptom levels at the beginning of treatment accounted for 11.4% of the variance in treatment outcome (Model 2). The final model (Model 5) including symptom level, engagement persistence, and enrolled course explained 64.3% of variance in treatment outcome. No effect on treatment outcome could be found for the intensity of patient engagement, the written interaction with the clinician and written clinician engagement. There was also a significant effect of the type of course patients completed, with the depression course yielding higher symptom improvements than the mixed anxiety/depression course and the social anxiety/depression course. None of the control variables contributed significantly. The variance accounted for by the inclusion of predictors in the model served as an indicator of the influence of a particular predictor or predictors introduced in different models. In all models, the addition of control and predictor variables successively decreased the variance in slopes compared to Model 1. The highest decrease in slope variance was found for the inclusion of engagement persistence into the model, leading to an overall increase in the explained variance in symptom change of 44.3% (~ *R*^2^) compared to Model 1.

**Table 4:** Results of multilevel growth curve analysis: predictors of symptom change during therapy.

**Models**	**Model 0**	**Model 1**	**Model 2**	**Model 3**	**Model 4**	**Model 5**	**Model 0**	**Model 1**	**Model 2**	**Model 3**	**Model 4**	**Model 5**
	**Est**	**Est**	**Est**	**Est**	**Est**	**Est**	**Est**	**Est**	**Est**	**Est**	**Est**	**Est**
***Fixed effects***	**PHQ-9 (*****N*** = **374)**						**GAD-7 (*****N*** = **313)**					
Intercept	12.828 (0.267)^**^	12.798 (0.245)^**^	6.882 (0.368)^**^	8.031 (0.982)^**^	8.034 (0.739)^**^	7.501 (0.591)^**^	8.765 (0.239)^**^	8.751 (0.227)^**^	6.119 (0.189)^**^	6.089 (0.626)^**^	6.117 (0.192)^**^	6.259 (0.253)^**^
Time	−0.297 (0.018)^**^	−0.283 (0.021)^**^	−0.101 (0.041)^*^	0.076 (0.087)	−0.024 (0.075)	0.035 (0.058)	−0.197 (0.016)^**^	−0.192 (0.019)^**^	−0.093 (0.024)^**^	0.010 (0.080)	−0.070 (0.024)^**^	−0.059 (0.032)
Symptom level			7.731 (0.421)^**^	7.765 (0.419)^**^	7.787 (0.416)^**^	7.603 (0.440)^**^			6.321 (0.292)^**^	6.307 (0.296)^**^	6.333 (0.291)^**^	6.241 (0.298)
Age				−0.377 (0.185)^*^	−0.338 (0.185)					−0.234 (0.151)		
Gender				0.252 (0.359)						0.223 (0.151)		
EP					−0.220 (0.181)	−0.400 (0.177)^*^					−0.046 (0.169)	−0.030 (0.168)
EI					0.138 (0.258)						−0.303 (0.111)^**^	−0.269 (0.110)^*^
WI					0.927 (0.262)^**^						−0.054 (0.174)	
CE					−0.637 (0.251)^*^						0.287 (0.132)	
Enrolled course						n.s.						−0.205 (0.301)∇
Time × Symptom Level			−0.237 (0.047)^**^	−0.234 (0.047)^**^	−0.221 (0.042)^**^	−0.270 (0.043)^**^			−0.236 (0.037)^**^	−0.244 (0.038)^**^	−0.239 (0.037)^**^	−0.242 (0.038)^**^
Time × Age				−0.052 (0.021) ^*^	−0.30 (0.019)					−0.028 (0.019)		
Time × Gender				0.002 (0.040)						−0.004 (0.037)		
Time × EP					−0.156 (0.018)^**^	−0.175 (0.017)^**^					−0.127 (0.021)^**^	−0.128 (0.021)^**^
Time × EI					0.020 (0.027)						0.039 (0.014)^**^	0.039 (0.014)^**^
Time × WI					−0.028 (0.027)						−0.017 (0.022)	
Time × CE					−0.034 (0.026)						−0.008 (0.024)	
Time × Enrolled course						^**^♢						−0.027 (0.038)^**^∇
Residual	9.692 (0.408)^**^	7.697 (0.388)^**^	7.601 (0.378)^**^	7.610 (0.379)^**^	7.609 (0.380)^**^	7.611 (0.380)^**^	5.935 (0.260)^**^	4.983 (0.258)^**^	4.854 (0.230)	4.860 (0.231)^**^	4.627 (0.218)^**^	4.643 (0.219)^**^
Variance in intercept	20.607 (1.742)^**^	17.611 (1.679)^**^	7.199 (0.889)^**^	7.081 (0.885)^**^	6.729 (0.874)^**^	7.070 (0.895)^**^	13.483 (1.220)	12.456 (1.311)^**^	2.938 (0.538)	2.958 (0.542)^**^	2.867 (0.526)^**^	2.891 (0.528)^**^
Variance in slopes	-	0.070 (0.012)^**^	0.062 (0.011)^**^	0.060 (0.011)^**^	0.029 (0.009)^**^	0.025 (0.009)^**^	-	0.044 (0.009)^**^	n.c.	n.c.	n.c.	n.c.
AIC	8557.05	8483.41	8230.88	8231.36	8021.4	8004.28	7042.69	7006.22	6713.42	6724.01	6546.91	6539.38
~*R*^2^ in slopes		-	0.114	0.143	0.586	0.643		-	n.c.	n.c.	n.c.	n.c.

EP, Engagement Persistency; EI, Engagement Intensity; WI, Written Interaction with Clinician; CE, Clinician Engagement. PHQ-9: ♢time × course_id 83[−1.050 (0.579)]^**^; time × course_id 94 [−0.473 (0.523)]; time × course_id 99 (0). GAD-7: ∇course_id 93^**^; course_id 94 (0). Mixed models for GAD-7 scores could not be converged, and parameters in the models not computed.

### 3.2 Anxiety symptoms

#### 3.2.1 Symptom change between MHC completion and first point of measurement

In the context of the MHC, the GAD-7 questionnaire is only administered to patients who score above 2 on questions 3 and 4 of the PHQ-4. Consequently, individuals who participated in an anxiety program, but scored low on these questions in the initial assessment, resulting in an incomplete GAD-7 assessment, were excluded from the subsequent analyses. Within the remaining group of patients (*N* = 258), significant improvements in symptoms were observed at the outset of treatment, with a notable reduction in anxiety symptoms observed between the completion of the MHC assessment and the initial evaluation following the commencement of the online intervention in module 2 (*t-value* = 15.985, *df* = 257; *p* < 0.001). Across the entire patient sample, effect sizes in the initial treatment phase were large for the GAD-7, with a Cohen's *d* of 1.00.

#### 3.2.2 Symptom change during program completion

Results from multilevel models for the GAD-7 during treatment are shown in Table 3. In patients with a GAD-7 score above the cut-off at the beginning of treatment, convergence could not be reached when a random effect of time was added. Statistically significant improvements during treatment were found in anxiety scores for both subclinical (*n* = 183) and clinical (*n* = 130) symptom levels. For the total score of the GAD-7, the intercept was estimated at 6.11 for the subclinical sample, and 12.43 for the clinical sample. The rates of change were estimated to be an average decrease of 0.32 points per module for clinical symptom levels and 0.09 points per module for subclinical symptom levels. This equals an estimated overall mean change of 3.2 points for clinical and 0.9 points for subclinical pre-treatment symptom levels if patients completed all 10 modules. In the program phase, patients completing an anxiety program and starting with clinical symptom levels displayed a large effect in the GAD-7 (Cohen's *d* = 0.60, classified as a medium effect). To reach a change in anxiety symptoms ≥ RCI of 3.13 based on a general population sample (Löwe et al., 2008), completion of 6.4 modules would have been necessary for Braive patients starting treatment above cut-off. Patients completing an anxiety program and scoring below the cut-off at the beginning of module completion showed very small effects (Cohen's *d* = 0.17).

#### 3.2.3 User metrics predictors of symptom change

The outcomes of the multilevel models employed to estimate and forecast changes in GAD-7 scores are detailed in Table 4. Notably, we observed a significant inverse relationship between the initial symptom level and the patients' engagement persistence in the developmental trajectory of GAD-7 scores. Specifically, both higher initial symptom levels at the start of treatment and greater patient engagement persistence corresponded to more substantial alterations in anxiety levels over time. The intensity of patient engagement had a significant positive effect on the developmental trajectory and therefore a negative association with outcome. Both the written interaction with the clinician as assessed by the total number of words and messages sent from patient to clinician and the clinician's engagement as assessed by the number and length of clinician messages sent to the patient did not contribute significantly to treatment outcome. There was also a significant effect on the type of course patients completed, with the anxiety program yielding higher symptom improvements than the mixed anxiety and depression program. The influence of each particular predictor added to the models measured by the variance in slopes could not be computed. Looking at Models 2 to 5, adding predictor variables successively decreased the AIC compared to Model 1, indicating a better model fit when including symptom levels, engagement persistence, and enrolled courses as predictors of change. None of the control variables were found to have a significant effect on treatment outcome.

## 4 Discussion

### 4.1 Main findings

The present study rested on the monitoring of the therapy process in patients attending an internet-delivered treatment program in a naturalistic setting. Our goal was to explore the relationship between user engagement and treatment outcomes for both subclinical and clinical levels of depression and anxiety in a guided iCBT intervention under real-life conditions.

Overall, our results align with several previous studies demonstrating the effectiveness of guided iCBT interventions targeting depression and anxiety symptoms in real-world settings (Etzelmueller et al., 2020), though it is important to note that the symptom severity in our sample was on average moderate, rather than severe. While we observed significant symptom improvements across the treatment process, particularly for those with higher initial symptom levels, the findings underscore the complexity of treatment responsiveness across different severity levels. Our study also highlights the role of user engagement, where greater persistence in completing modules and activities was associated with better outcomes, suggesting that engagement is a key factor in maximizing treatment benefits (Donkin et al., 2011; Christensen et al., 2002; Enrique et al., 2019).

However, the level of program engagement, as measured by the number and duration of log-ins, was not linked to any improvement. A greater number of logins and minutes spent on the platform were even found to be slightly negatively associated with outcomes in anxiety patients. Again, one could argue that the number of logins and the total session length are not *per se* indicative of “doing what is useful." In anxiety treatment, for instance, one of the main effective ingredients of CBT is exposure, which mainly happens outside of the platform. However, activity outside of the platform is not covered by the log data. A more accurate indicator for comprehensively engaging in content may be actual task completion over a longer treatment period, as assessed in our factor engagement persistence. In line with our findings, Donkin et al. (2013) found no significant difference in the number of log-ins between patients who achieved clinically significant change and those who did not. However, their study did show a significant variation in the total time spent in the program between these groups. The authors suggested that the correlation between older age and more time spent online may be due to lower computer proficiency or slower cognitive processing rather than higher engagement. Interestingly, our results mirrored this finding, as time spent on the platform increased steadily with age (16–19 y: M = 193.37, SD = 150.42; 20–25 y: M = 501.61, SD = 345.20; 26–34 y: M = 569.58, SD = 572.06; 35–44 y: M = 599.02, SD = 609.13; 45–65 y: M = 806.72, SD = 780.79; 65+ y: M = 2130.44, SD = 1658.46), but age did not significantly affect treatment outcome. Given that we were unable to separate time spent in video calls from overall time on the platform, this age-related increase may also reflect a greater reliance on therapist contact among older adults, suggesting that the correlation between age and time online could partly be an expression of increased need or preference for synchronous communication with a therapist.

Our analysis showed that the quantity of written communication between patient and clinician did not predict treatment outcomes. However, our study assessed only the volume of written communication, not the therapeutic quality of the therapist feedback, nor the frequency and length of video communication. As such, drawing the conclusion that therapist support is unimportant for treatment success would be unwarranted. With a few notable exceptions (Titov et al., 2009; Berger et al., 2011), previous research suggests that guided self-help treatments generally achieve better results than unguided ones (Spek et al., 2007).

The degree of symptom improvement observed early on, even before exposure to the iCBT content, was somewhat unexpected and particularly striking for anxiety symptoms. We can speculate that it might have been related to other factors such as the decision to commence treatment, the therapeutic effects of assessment in itself, or receiving an individually tailored recommendation after completing the MHC. Our results are in line with a recent paper examining symptom improvement during an 8-week online treatment for depression and anxiety (Bisby et al., 2023). Their findings revealed a swift and substantial reduction in symptoms at the early stages of treatment, regardless of the diagnosis or the specific outcome measure used.

With regards to other potential predictors of change, patients with higher symptom levels showed a greater symptom reduction during treatment, and this held true for both depression and anxiety symptoms. This result is in accordance with studies indicating that patients starting off with higher initial symptom levels show greater symptom improvement over the course of treatment (Erbe et al., 2017; González-Robles et al., 2021). One must keep in mind, though, that in the current sample, even patients with clinical symptom levels on average did not actually have high severity levels pre-treatment (PHQ-9 = 15.55 (3.76); high severity: ≥20); GAD-7 = 13.43 (2.87); high severity: ≥15) and thus, our findings could be due to a statistical artifact. In our sample, patients meeting clinical criteria for depression showed average pre-treatment scores in the moderate range (PHQ-9: M = 15.55, SD = 3.76; GAD-7: M = 13.43, SD = 2.87), falling below thresholds typically used to define high symptom severity (PHQ-9 ≥20; GAD-7 ≥15). As such, the observed pattern may reflect a regression to the mean effect rather than true variation in treatment responsiveness across severity levels.

### 4.2 Strengths and limitations

One of the key strengths of the present study is the use of multilevel modeling (MLM) to analyze the repeated use of process- and outcome measures throughout treatment and operationalize patient change. Whereas, a pre-post research design coupled with ANOVA as a statistical method assumes that all patients benefit equally from a given treatment, MLM separates within- and between-patient variance components of the process-outcome relation (Kahn and Schneider, 2013) and thus allowed us to investigate within-group variability in symptom change and its relation to user engagement across the entire treatment trajectory.

Another potential strength lies in its monitoring of a sizeable cohort of patients receiving routine psychotherapeutic care, offering valuable insights into therapy progress and overall outcomes in a naturalistic setting. In contrast, patient selection is stricter in most research trials, and patients with subclinical symptom scores, dual diagnoses, or high levels of psychopathology tend to get excluded, which may compromise external validity. Furthermore, trial participants might experience advantages from assessment effects or in-person interactions that are not directly connected to the intervention itself. This bias is ruled out in a real-world context, and the somewhat smaller improvements obtained may provide more realistic estimates of effect sizes in real-world settings (Leichsenring and Rüger, 2004). Hence, the naturalistic design provided strong external validity by closely mirroring the real-world conditions of a standardized internet-based psychotherapy program.

The flipside, however, is that the collection of log data was carried out independently of research considerations, which means that crucial parameters, such as the frequency and duration of video calls with a clinician, were not available as a separate metric, but are confounded with time spent on the platform in our analyses. Higher “platform use" may therefore actually reflect more therapist contact, not necessarily more self-guided engagement with modules. As a result, even though video calls were an integral part of treatment, we were unable to assess their association with outcomes or their interaction with other predictors of change. For instance, a lower number of written messages may indicate a higher frequency of video calls, as clinician feedback is given through either method of support, or more or longer video calls with a therapist may imply more complex patients or patients who are off track. Following this reasoning, more time spent on the platform and unfavorable treatment trajectories would be confounded. Consequently, without distinguishing between video and chat, we were unable to draw conclusions about how different modes of therapist support (chat vs. video) may differentially impact treatment outcomes. Additionally, the user metrics were recorded as total scores across completed modules. While this provides a measure of the overall therapy dose, it lacks detail on how usage varied over time and how different features of the intervention were utilized. Moreover, the assumption that module and activity completion best reflect meaningful engagement may be overly simplistic. It is possible that some patients disengaged not due to lack of motivation or commitment, but because they found the program unhelpful or misaligned with their individual needs. In such cases, completed modules may indicate persistence rather than therapeutic value or satisfaction. Furthermore, our focus on immediate treatment outcomes limits our understanding of whether symptom reductions were sustained over time, or whether patterns of engagement predicted longer-term benefits. This limitation, along with the lack of granular engagement data, underscores the need for future research that includes follow-up assessments and more nuanced, patient-centered engagement metrics.

Some methodological issues also need mentioning. Firstly, the design did not include experimental manipulation or a control condition. Consequently, conclusions about cause and effect are not possible, as other factors or explanations may have contributed to the observed relationship between user metrics and outcomes. Secondly, linear mixed models for GAD scores could not be converged, and parameters for random estimates in those models were thus not computed. In the spirit of exploration, we tried to compute alternative models by removing the random time effect, but that was of no avail concerning converging. We concluded that the models could not be computed due to insufficient variability in slopes. However, since we were interested in the overall association between user metrics and symptom change during treatment, we included these analyses nevertheless, but results need to be treated with some caution. Thirdly, the impressive ~ *R*^2^ value for persistence of engagement should be considered tentatively. Several methods have been proposed in statistical literature to calculate ~ *R*^2^ for linear mixed models (LMM) (Snijders and Bosker, 1994; Xu, 2003; Gelman and Pardoe, 2006; Edwards et al., 2008; Nakagawa and Schielzeth, 2013). However, the approaches used to define ~*R*^2^ for LMM are very divergent, and all of them come with certain problematic issues in ~ *R*^2^ estimation (for an overview, see Jaeger et al., 2017). Common challenges in extending ~*R*^2^ to LMM and general linear mixed models include negative estimated ~*R*^2^ values, reduced ~*R*^2^ values with additional fixed predictors, and the need for advanced statistical expertise to implement ~*R*^2^ calculation methods. Consequently, agreement on the definition of R2 in LMM remains elusive. We used two methods of ~ *R*^2^ computation:


$$\begin{array}{l} 1.\left(\text{var slopes null }-\text{ var slopes pred}\right)/\text{var slopes null}\\ 2.\text{t}2/\left(\text{t}2+\text{df}\right) \end{array}$$

As you can see in Tables 4, 5, these methods reached different results, but point in the same direction, i.e., that engagement persistence is strongly related to treatment outcome.

**Table 5:** Explained variance with step-by-step analysis, using t-score and df to compute ~*R*^2^.

***Fixed effects***	**Symptom level**	**Engagement persistency**	**Engagement intensity**	**Written interaction**	**Clinician Engagement**	**Symptom level**	**Engagement persistency**	**Engagement intensity**	**Written interaction**	**Clinician Engagement**
	**Est**	**Est**	**Est**	**Est**	**Est**	**Est**	**Est**	**Est**	**Est**	**Est**
	**PHQ-9**					**GAD-7**				
Intercept	6.882 (0.368)^**^	12.752 (0.250)^**^	12.810 (0.250)^**^	12.833 (0.249)^**^	12.784 (0.246)^**^	6.119 (0.189)^**^	8.712 (0.233)^**^	8.760 (0.228)^**^	8.727 (0.229)^**^	8.748 (0.228)^**^
Time	−0.101 (0.041)^*^	−0.297 (0.019)^**^	−0.286 (0.021)^**^	−0.289 (0.021)^**^	−0.288 (0.020)^**^	−0.093 (0.024)^**^	−0.167 (0.018)^**^	−0.196 (0.019)^**^	−0.190 (0.019)^**^	−0.191 (0.019)^**^
Covariate	7.731 (0.421)^**^	−0.202 (0.239)	0.184 (0.365)	0.501 (0.284)	−0.073 (0.257)	6.321 (0.292)^**^	0.011 (0.256)	−0.266 (0.175)	0.028 (0.201)	0.039 (0.217)
Time × Coavariate	−0.237 (0.047)^**^	−0.173 (0.018)^**^	−0.022 (0.031)	−0.064 (0.024)^**^	−0.095 (0.021)^**^	−0.236 (0.037)^**^	−0.131 (0.020)^**^	0.049 (0.015)^**^	−0.010 (0.017)	−0.012 (0.018)
t (time × cov)	−5.005	−9.613	−0.733	−2.697	−4.488	−6.319	−6.565	3.343	−0.599	−0.671
df (time × cov)	396.97	410.953	360.364	367.341	383.584	1361	331.424	305.033	308.175	314.61
~*R*^2^ (t^2^/(t^2^+df))	−0.067	−0.29	−0.001	−0.02	−0.055	−0.03	−0.149	−0.037	−0.001	−0.001

^*^p < 0.05; ^**^p < 0.01.

Lastly, the last-observation-carried-forward (LOCF) method might underestimate the effectiveness of the intervention, as certain studies have indicated that individuals who experience symptom improvements may discontinue their participation (Postel et al., 2010; Lawler et al., 2021). However, the LOCF approach assumes no improvement in such cases.

### 4.3 Clinical implications and future directions

In our sample, all patients on average exhibited a degree of involvement that was associated with some kind of positive outcomes. Additionally, we observed that the continued use of the platform, indicated by the completion of modules and activities, enhanced treatment effectiveness. Interestingly, the overall duration spent on the platform, frequency of logins, and the level of written communication between patients and clinicians did not show a correlation with improvements in symptoms. These results hold importance as they imply that both iCBT providers and clinicians have the opportunity to enhance treatment outcomes by promoting exposure to and active engagement with therapeutic material.

On the one hand, iCBT providers should ensure that their platform is user-friendly, intuitive, and visually appealing to facilitate ease of navigation and engagement (McCall et al., 2021). Providing personalized treatment plans and content based on individual needs, preferences, and progress assessments may also increase relevance and user engagement (Mukhiya et al., 2020). Incorporating interactive elements such as gamification techniques, exercises, progress trackers, multimedia content and social support features may further increase active participation in the material. Additionally, offering timely and supportive communication, including reminders, prompts, and feedback, can encourage adherence in the therapy process (Dennison et al., 2013). The combination of log data collection and Ecological Momentary Assessment (EMA) could be used to gain a better understanding of adherence to tasks that need to be completed outside of the platform (e.g., behavioral activation, exposure) and to guide tailored recommendations for identifying which skill areas could offer the greatest therapeutic benefits for individual patients (Webb et al., 2022).

Clinicians, on the other hand, should provide guidance and support to patients in navigating the therapeutic material, clarifying concepts, and addressing any concerns or questions that may arise. Monitoring patient progress, providing feedback on engagement levels, identifying and addressing barriers to engagement (such as technical difficulties), and offering encouragement and reinforcement may also help sustain motivation (Lutz et al., 2022). Furthermore, collaboratively setting treatment goals with patients and involving them in decision-making regarding therapeutic content and strategies has been shown to enhance ownership and commitment (Pihlaja et al., 2018). Lastly, therapists should be flexible in adjusting treatment plans and therapeutic approaches based on patient preferences, feedback, and changing needs throughout therapy.

In this context, it is important for future work to investigate the distinct effects of different communication modalities-specifically, written (chat-based) vs. video-based therapist feedback. Since both forms of support can serve similar functions but may be differentially suited to patient characteristics or clinical needs, understanding their relative impact on outcomes could provide valuable insights for optimizing therapist involvement in guided iCBT. The current study did not differentiate between video call time and other forms of engagement (e.g., written communication), which makes it difficult to assess the specific contribution of therapist video interactions to treatment outcomes. This limitation is particularly important as video calls may have unique therapeutic effects, which could impact engagement and symptom reduction differently than chat-based support. For instance, video calls may foster a stronger therapeutic alliance or provide more personalized feedback, influencing treatment outcomes in ways that written communication may not. Without this differentiation, we cannot fully evaluate the potential of video-based interventions or their interaction with other factors such as patient characteristics and treatment adherence.

An additional area for further investigation involves the early symptom improvement observed in this study–particularly for anxiety–which occurred even before participants began engaging with the core iCBT content. This early change may reflect factors such as the therapeutic effect of assessment, the motivational impact of deciding to start treatment, or the benefit of receiving a personalized treatment recommendation after completing the Mental Health Check. Future studies could explore these possibilities in more depth, for instance by employing a Solomon four-group design to disentangle assessment effects from intervention effects and better understand the mechanisms driving early change.

Future research should also examine how the above-mentioned factors influence adherence to internet-delivered interventions. Advanced methods of treatment personalization, such as machine-learning models, could be utilized to develop and test algorithms and adaptive systems that dynamically adjust treatment content and delivery based on user data. While the study included multiple therapists, it did not account for differences in therapeutic style, feedback quality, or the patient-therapist relationship – all of which may have influenced engagement and outcomes. Future work should therefore examine how patient characteristics interact with therapist features, including individual preferences and needs. Existing literature suggests that these factors can significantly impact treatment engagement and outcomes (Williams et al., 2016; Monzani et al., 2020). The integration of emerging technologies, such as artificial intelligence, virtual reality, and wearable devices, should be explored and end-users involved to develop and refine online treatment platforms and interventions. It will also be essential to examine factors influencing the successful implementation and dissemination of guided iCBT interventions in real-world settings, including organizational readiness, clinician attitudes and training needs, and reimbursement policies. Understanding usage patterns–both in terms of adherence over time and frequently visited treatment elements–could offer valuable insights for clinical practice, particularly in identifying critical points that may help explain potential dropout risks. While the focus on symptom reduction remains central, future studies should also consider including broader outcome measures, such as patients' self-perceived changes in personal resources like self-efficacy, resilience, or coping capacity. These indicators could offer a more comprehensive understanding of therapeutic progress and highlight how individuals perceive their ability to manage challenges beyond the scope of symptom alleviation alone.

## 5 Conclusion

This study reinforces previous findings that greater consumption of therapeutic content is linked to better outcomes in internet-delivered psychotherapy for anxiety and depression. Persistence in engaging with the platform emerged as a key factor, highlighting the need to promote sustained exposure to treatment material through both thoughtful program design and active clinical support. Future research should explore how different modes of therapist communication may differentially impact outcomes, and how these modalities align with individual patient needs to further optimize engagement and treatment effectiveness.

## Data Availability

The data analyzed in this study is subject to the following licenses/restrictions: data contains sensitive information. Requests to access these datasets should be directed to Karin Hammerfald, karin.hammerfald@psykologi.uio.no.

## Footnotes

^1^PHQ-9: assessed at beginning of the first module; GAD-7: assessed at beginning of the second module. This means that information about GAD-7 levels upon the start of the online program is missing.

## References

[B1] Andersson G. Carlbring P. Rozental A. (2019). Response and remission rates in internet-based cognitive behavior therapy: an individual patient data meta-analysis. Front. Psychiatry 10, 749–749. 10.3389/fpsyt.2019.00749 31708813 PMC6823683

[B2] Andrews G. Basu A. Cuijpers P. Craske M. McEvoy P. English C.. (2018). Computer therapy for the anxiety and depression disorders is effective, acceptable and practical health care: an updated meta-analysis. J. Anxiety Disord. 55, 70–78. 10.1016/j.janxdis.2018.01.001 29422409

[B3] Batterham P. J. Mackinnon A. J. Christensen H. (2015). The panic disorder screener (padis): development of an accurate and brief population screening tool. Psychiatry Res. 228, 72–76. 10.1016/j.psychres.2015.04.016 25956758

[B4] Beard C. Björgvinsson T. (2014). Beyond generalized anxiety disorder: Psychometric properties of the gad-7 in a heterogeneous psychiatric sample. J. Anxiety Disord. 28, 547–552. 10.1016/j.janxdis.2014.06.002 24983795

[B5] Berger T. Hämmerli K. Gubser N. Andersson G. Caspar F. (2011). Internet-based treatment of depression: a randomized controlled trial comparing guided with unguided self-help. Cogn. Behav. Ther. 40, 251–266. 10.1080/16506073.2011.616531 22060248

[B6] Besèr A. Sorjonen K. Wahlberg K. Peterson U. Nygren Å. Åsberg M. (2014). Construction and evaluation of a self rating scale for stress-induced exhaustion disorder, the karolinska exhaustion disorder scale. Scand. J. Psychol. 55, 72–82. 10.1111/sjop.12088 24236500 PMC4235404

[B7] Bisby M. A. Scott A. J. Fisher A. Gandy M. Hathway T. Heriseanu A. I.. (2023). The timing and magnitude of symptom improvements during an internet-delivered transdiagnostic treatment program for anxiety and depression. J. Consult. Clin. Psychol. 91, 95–111. 10.1037/ccp0000761 36201813

[B8] Bryk A. S. (1992). Hierarchical Linear Models: Applications and Data Analysis Methods. Newbury Park: Sage.

[B9] Bullis J. R. Boettcher H. Sauer-Zavala S. Farchione T. J. Barlow D. H. (2019). What is an emotional disorder? a transdiagnostic mechanistic definition with implications for assessment, treatment, and prevention. Clin. Psychol. 26:e12278-n/a. 10.1111/cpsp.12278

[B10] Cameron I. M. Crawford J. R. Lawton K. Reid I. C. (2008). Psychometric comparison of phq-9 and hads for measuring depression severity in primary care. Br. J. Gen. Pract. 58, 32–36. 10.3399/bjgp08X263794 18186994 PMC2148236

[B11] Carlbring P. Andersson G. Cuijpers P. Riper H. Hedman-Lagerlöf E. (2018). Internet-based vs. face-to-face cognitive behavior therapy for psychiatric and somatic disorders: an updated systematic review and meta-analysis. Cogn. Behav. Ther. 47, 1–18. 10.1080/16506073.2017.1401115 29215315

[B12] Chalder T. (1996). Insomnia: psychological assessment and management. by C. M. Morin. guildford press: New york. 1993. Psychol. Med. 26, 1096–1097. 10.1017/S0033291700035467

[B13] Christensen H. Griffiths K. M. Farrer L. (2009). Adherence in internet interventions for anxiety and depression: systematic review. J. Med. Internet Res. 11:e13. 10.2196/jmir.1194 19403466 PMC2762797

[B14] Christensen H. Griffiths K. M. Korten A. (2002). Web-based cognitive behavior therapy: analysis of site usage and changes in depression and anxiety scores. J. Med. Internet Res. 4, e3-e3. 10.2196/jmir.4.1.e3 11956035 PMC1761927

[B15] Cohen J. (1988). Statistical Power Analysis for the Behavioral Sciences. Hillsdale, NJ: Laurence Erlbaum.

[B16] Cohen S. Kamarck T. Mermelstein R. (1983). A global measure of perceived stress. J. Health Soc. Behav. 24, 385–396. 10.2307/2136404 6668417

[B17] Connor K. M. Kobak K. A. Churchill L. E. Katzelnick D. Davidson J. R. (2001). Mini-spin: a brief screening assessment for generalized social anxiety disorder. Depress. Anxiety 14, 137–140. 10.1002/da.1055 11668666

[B18] Couper M. P. Alexander G. L. Zhang N. Little R. J. A. Maddy N. Nowak M. A.. (2010). Engagement and retention: measuring breadth and depth of participant use of an online intervention. J. Med. Internet Res. 12:e52. 10.2196/jmir.1430 21087922 PMC3056524

[B19] Dear B. Staples L. Terides F.ogliati, V. Sheehan J. Johnston L. Kayrouz R.. (2016). Transdiagnostic versus disorder-specific and clinician-guided versus self-guided internet-delivered treatment for social anxiety disorder and comorbid disorders: a randomized controlled trial. J. Anxiety Disord. 42, 30–44. 10.1016/j.janxdis.2016.05.004 27261562

[B20] Dennison L. Morrison L. Conway G. Yardley L. (2013). Opportunities and challenges for smartphone applications in supporting health behavior change: qualitative study. J. Med. Internet Res. 15:e2583. 10.2196/jmir.2583 23598614 PMC3636318

[B21] Donkin L. Christensen H. Naismith S. L. Neal B. Hickie I. B. Glozier N. (2011). A systematic review of the impact of adherence on the effectiveness of e-therapies. J. Med. Internet Res. 13:e52. 10.2196/jmir.1772 21821503 PMC3222162

[B22] Donkin L. Hickie I. B. Christensen H. Naismith S. L. Neal B. Cockayne N. L.. (2013). Rethinking the dose-response relationship between usage and outcome in an online intervention for depression: randomized controlled trial. J. Med. Internet Res. 15, e231-e231. 10.2196/jmir.2771 24135213 PMC3806549

[B23] Edwards L. J. Muller K. E. Wolfinger R. D. Qaqish B. F. Schabenberger O. (2008). An r2 statistic for fixed effects in the linear mixed model. Stat. Med. 27, 6137–6157. 10.1002/sim.3429 18816511 PMC2587505

[B24] Enrique A. Palacios J. E. Ryan H. Richards D. (2019). Exploring the relationship between usage and outcomes of an internet-based intervention for individuals with depressive symptoms: Secondary analysis of data from a randomized controlled trial. J. Med. Internet Res. 21, e12775-e12775. 10.2196/12775 31373272 PMC6694731

[B25] Erbe D. Eichert H.-C. Rietz C. Ebert D. (2016). Interformat reliability of the patient health questionnaire: Validation of the computerized version of the phq-9. Internet Interv. 5, 1–4. 10.1016/j.invent.2016.06.006 30135800 PMC6096192

[B26] Erbe D. Eichert H.-C. Riper H. Ebert D. D. (2017). Blending face-to-face and internet-based interventions for the treatment of mental disorders in adults: systematic review. J. Med. Internet Res. 19:e306. 10.2196/jmir.6588 28916506 PMC5622288

[B27] Etzelmueller A. Vis C. Karyotaki E. Baumeister H. Titov N. Berking M.. (2020). Effects of internet-based cognitive behavioral therapy in routine care for adults in treatment for depression and anxiety: Systematic review and meta-analysis. J. Med. Internet Res. 22:e18100. 10.2196/18100 32865497 PMC7490682

[B28] Eysenbach G. (2005). The law of attrition. J. Med. Internet Res. 7:e11. 10.2196/jmir.7.1.e11 15829473 PMC1550631

[B29] Fuhr K. Schröder J. Berger T. Moritz S. Meyer B. Lutz W.. (2018). The association between adherence and outcome in an internet intervention for depression. J. Affect. Disord. 229, 443–449. 10.1016/j.jad.2017.12.028 29331706

[B30] Gebauer L. LaBrie R. Shaffer H. J. (2010). Optimizing dsm-iv-tr classification accuracy: a brief biosocial screen for detecting current gambling disorders among gamblers in the general household population. Can. J. Psychiatry 55, 82–90. 10.1177/070674371005500204 20181303

[B31] Gelman A. Pardoe I. (2006). Bayesian measures of explained variance and pooling in multilevel (hierarchical) models. Technometrics 48, 241–251. 10.1198/00401700500000051712611515

[B32] González-Robles A. Suso-Ribera C. Díaz-García A. García-Palacios A. Castilla D. Botella C. (2021). Predicting response to transdiagnostic icbt for emotional disorders from patient and therapist involvement. Internet Interv. 25:100420. 10.1016/j.invent.2021.100420 34401379 PMC8350608

[B33] Hall R. C. (1995). Global assessment of functioning: a modified scale. Psychosomatics 36, 267–275. 10.1016/S0033-3182(95)71666-8 7638314

[B34] Hamer R. M. Simpson P. M. (2009). Last observation carried forward versus mixed models in the analysis of psychiatric clinical trials. Am. J. Psychiatry 166, 639–641. 10.1176/appi.ajp.2009.09040458 19487398

[B35] Ito M. Nakajima S. Fujisawa D. Miyashita M. Kim Y. Shear M. K.. (2012). Brief measure for screening complicated grief: reliability and discriminant validity. PLoS ONE 7:e31209. 10.1371/journal.pone.0031209 22348057 PMC3279351

[B36] Jaeger B. C. Edwards L. J. Das K. Sen P. K. (2017). An r 2 statistic for fixed effects in the generalized linear mixed model. J. Appl. Stat. 44, 1086–1105. 10.1080/02664763.2016.1193725

[B37] Kahn J. H. Schneider W. J. (2013). It's the destination and it's the journey: using multilevel modeling to assess patterns of change in psychotherapy: multilevel modeling. J. Clin. Psychol. 69, 543–570. 10.1002/jclp.21964 23348510

[B38] Kroenke K. Spitzer R. L. (2002). The phq-9: a new depression diagnostic and severity measure. Psychiatric Ann. 32:509–515. 10.3928/0048-5713-20020901-06

[B39] Kroenke K. Spitzer R. L. Williams J. Löwe B. (2010). The patient health questionnaire somatic, anxiety, and depressive symptom scales: a systematic review. Gen. Hosp. Psychiatry 32, 345–359. 10.1016/j.genhosppsych.2010.03.006 20633738

[B40] Kroenke K. Spitzer R. L. Williams J. B. Löwe B. (2009). An ultra-brief screening scale for anxiety and depression: the phq-4. Psychosomatics 50, 613–621. 10.1176/appi.psy.50.6.613 19996233

[B41] Langbehn D. Pfohl B. Reynolds S. Clark L. Battaglia M. Bellodi L.. (1999). The iowa personality disorder screen: development and preliminary validation of a brief screening interview. J. Pers. Disord. 13, 75–89. 10.1521/pedi.1999.13.1.75 10228929

[B42] Lawler K. Earley C. Timulak L. Enrique A. Richards D. (2021). Dropout from an internet-delivered cognitive behavioral therapy intervention for adults with depression and anxiety: qualitative study. JMIR Form. Res. 5:e26221. 10.2196/26221 34766909 PMC8663602

[B43] Leichsenring F. Rüger U. (2004). Psychotherapeutische behandlungsverfahren auf dem prüfstand der evidence based medicine (EBM): randomisierte kontrollierte studien vs. naturalistische studien – gibt es nur einen goldstandard? Zeitschrift für Psychosomatische Medizin und Psychotherapie 50, 203–217. 10.13109/zptm.2004.50.2.203 15146395

[B44] Löwe B. Decker O. Müller S. Brähler E. Schellberg D. Herzog W.. (2008). Validation and standardization of the generalized anxiety disorder screener (gad-7) in the general population. Med. Care 46, 266–274. 10.1097/MLR.0b013e318160d093 18388841

[B45] Löwe B. Unützer J. Callahan C. M. Perkins A. J. Kroenke K. (2004). Monitoring depression treatment outcomes with the patient health questionnaire-9. Med. Care 42, 1194–1201. 10.1097/00005650-200412000-00006 15550799

[B46] Lutz W. Deisenhofer A.-K. Rubel J. Bennemann B. Giesemann J. Poster K.. (2022). Prospective evaluation of a clinical decision support system in psychological therapy. J. Consult. Clin. Psychol. 90:90. 10.1037/ccp0000642 34166000

[B47] Mancuso S. G. Knoesen N. P. Castle D. J. (2010). The dysmorphic concern questionnaire: a screening measure for body dysmorphic disorder. Aust. N. Z. J. Psychiatry 44, 535–542. 10.3109/00048671003596055 20397781

[B48] Martin A. Rief W. Klaiberg A. Braehler E. (2006). Validity of the brief patient health questionnaire mood scale (phq-9) in the general population. Gen. Hosp. Psychiatry 28, 71–77. 10.1016/j.genhosppsych.2005.07.003 16377369

[B49] McCall H. C. Hadjistavropoulos H. D. Sundström C. R. F. (2021). Exploring the role of persuasive design in unguided internet-delivered cognitive behavioral therapy for depression and anxiety among adults: systematic review, meta-analysis, and meta-regression. J. Med. Internet Res. 23:e26939. 10.2196/26939 33913811 PMC8120424

[B50] McMillan D. Gilbody S. Richards D. (2010). Defining successful treatment outcome in depression using the phq-9: a comparison of methods. J. Affect. Disord. 127, 122–129. 10.1016/j.jad.2010.04.030 20569992

[B51] McNeely J. Wu L.-T. Subramaniam G. Sharma G. Cathers L. A. Svikis D.. (2016). Performance of the tobacco, alcohol, prescription medication, and other substance use (taps) tool for substance use screening in primary care patients. Ann. Intern. Med. 165, 690–699. 10.7326/M16-0317 27595276 PMC5291717

[B52] Monzani D. Vergani L. Pizzoli S. F. M. Marton G. Mazzocco K. Bailo L.. (2020). Sexism interacts with patient-physician gender concordance in influencing patient control preferences: Findings from a vignette experimental design. Appl. Psychol. Health Well-Being 12, 471–492. 10.1111/aphw.12193 31985173 PMC7384069

[B53] Morgan J. Reid F. Lacey H. (2000). The scoff questionnaire: assessment of a new screening tool for eating disorders. BMJ 319, 1467–1468. 10.1136/bmj.319.7223.1467 10582927 PMC28290

[B54] Mukhiya S. K. Wake J. D. Inal Y. Pun K. I. Lamo Y. (2020). Adaptive elements in internet-delivered psychological treatment systems: systematic review. J. Med. Internet Res. 22:e21066. 10.2196/21066 33245285 PMC7732710

[B55] Nakagawa S. Schielzeth H. (2013). A general and simple method for obtaining r2 from generalized linear mixed-effects models. Methods Ecol. Evol. 4, 133–142. 10.1111/j.2041-210x.2012.00261.x30239975

[B56] Nordh M. Wahlund T. Jolstedt M. Sahlin H. Bjureberg J. Ahlen J.. (2021). Therapist-guided internet-delivered cognitive behavioral therapy vs internet-delivered supportive therapy for children and adolescents with social anxiety disorder: a randomized clinical trial. JAMA Psychiatry 78, 705–713. 10.1001/jamapsychiatry.2021.0469 33978699 PMC8117054

[B57] Patel S. R. Cole A. Little V. Skritskaya N. A. Lever E. Dixon L. B.. (2019). Acceptability, feasibility and outcome of a screening programme for complicated grief in integrated primary and behavioural health care clinics. Family Practice 36, 125–131. 10.1093/fampra/cmy050 29860527

[B58] Pfender E. (2020). Mental health and COVID-19: implications for the future of telehealth. J. Patient Exp. 7, 433–435. 10.1177/2374373520948436 33062854 PMC7415938

[B59] Pihlaja S. Stenberg J.-H. Joutsenniemi K. Mehik H. Ritola V. Joffe G. (2018). Therapeutic alliance in guided internet therapy programs for depression and anxiety disorders-a systematic review. Internet Interv. 11, 1–10. 10.1016/j.invent.2017.11.005 30135754 PMC6084872

[B60] Plummer F.aye, M. Manea L. Trepel D. McMillan D. (2016). Screening for anxiety disorders with the gad-7 and gad-2: a systematic review and diagnostic metaanalysis. Gen. Hosp. Psychiatry 39, 24–31. 10.1016/j.genhosppsych.2015.11.005 26719105

[B61] Postel M. G. de Haan H. A. ter Huurne E. D. Becker E. S. de Jong C. A. J. (2010). Effectiveness of a web-based intervention for problem drinkers and reasons for dropout: randomized controlled trial. J. Med. Internet Res. 12:e68. 10.2196/jmir.1642 21163776 PMC3056532

[B62] Prins A. Bovin M. J. Smolenski D. J. Marx B. P. Kimerling R. Jenkins-Guarnieri M. A.. (2016). The primary care ptsd screen for dsm-5 (pc-ptsd-5): development and evaluation within a veteran primary care sample. J. Gen. Intern. Med. 31, 1206–1211. 10.1007/s11606-016-3703-5 27170304 PMC5023594

[B63] Rollman B. L. (2018). Effectiveness of online collaborative care for treating mood and anxiety disorders in primary care a randomized clinical trial (vol 75, pg 56, 2018). JAMA Psychiatry 75:104. 10.1001/jamapsychiatry.2017.3379 29117275 PMC5833533

[B64] Rosenström T. H. Saarni S. E. Saarni S. I. Tammilehto J. Stenberg J.-H. (2025). Efficacy and effectiveness of therapist-guided internet versus face-to-face cognitive behavioural therapy for depression via counterfactual inference using naturalistic registers and machine learning in finland: a retrospective cohort study. Lancet Psychiatry. 12, 189–197. 10.1016/S2215-0366(24)00404-8 39954684

[B65] Sieverink F. Kelders S. M. van Gemert-Pijnen J. E. (2017). Clarifying the concept of adherence to ehealth technology: Systematic review on when usage becomes adherence. J. Med. Internet Res. 19:e402. 10.2196/jmir.8578 29212630 PMC5738543

[B66] Singer J. D. Willett J. B. (2003). Applied Longitudinal Data Analysis: Modeling Change and Event Occurrence. Oxford: Oxford University Press. 10.1093/acprof:oso/9780195152968.001.0001

[B67] Snijders T. (2012). Multilevel Analysis : *An Introduction to Basic and Advanced Multilevel Modeling*. Thousand Oaks, CA: Sage.

[B68] Snijders T. A. Bosker R. J. (1994). Modeled variance in two-level models. Sociol. Methods Res. 22, 342–363. 10.1177/0049124194022003004

[B69] Spek V. Cuijpers P. Nyklíček I. Riper H. Keyzer J. Pop V. (2007). Internet-based cognitive behaviour therapy for symptoms of depression and anxiety: a meta-analysis. Psychol. Med. 37, 319–328. 10.1017/S0033291706008944 17112400

[B70] Spitzer R. L. Kroenke K. Williams J. B. Löwe B. (2006). A brief measure for assessing generalized anxiety disorder: the gad-7. Arch. Intern. Med. 166, 1092–1097. 10.1001/archinte.166.10.1092 16717171

[B71] Staples L. G. Dear B. F. Johnson B. Fogliati V. Gandy M. Fogliati R.. (2019). Internet-delivered treatment for young adults with anxiety and depression: Evaluation in routine clinical care and comparison with research trial outcomes. J. Affect. Disord. 256, 103–109. 10.1016/j.jad.2019.05.058 31170620

[B72] Steel Z. Marnane C. Iranpour C. Chey T. Jackson J. W. Patel V.. (2014). The global prevalence of common mental disorders: a systematic review and meta-analysis 1980–2013. Int. J. Epidemiol. 43, 476–493. 10.1093/ije/dyu038 24648481 PMC3997379

[B73] Terides M. D. Dear B. F. Fogliati V. J. Gandy M. Karin E. Jones M. P.. (2018). Increased skills usage statistically mediates symptom reduction in self-guided internet-delivered cognitive-behavioural therapy for depression and anxiety: a randomised controlled trial. Cogn. Behav. Ther. 47, 43–61. 10.1080/16506073.2017.1347195 28724338

[B74] The Lancet Global Health. (2020). Mental health matters. Lancet Global Health 8:e1352. 10.1016/S2214-109X(20)30432-0 33069297 PMC7561290

[B75] Titov N. Andrews G. Choi I. Schwencke G. Johnston L. (2009). Randomized controlled trial of web-based treatment of social phobia without clinician guidance. Aust. N. Z. J. Psychiatry 43, 913–919. 10.1080/00048670903179160

[B76] Titov N. Dear B. F. Johnston L. Lorian C. Zou J. Wootton B.. (2013). Improving adherence and clinical outcomes in self-guided internet treatment for anxiety and depression: randomised controlled trial. PLoS ONE 8:e62873. 10.1371/journal.pone.0062873 23843932 PMC3701078

[B77] Titov N. Dear B. F. McMillan D. Anderson T. Zou J. Sunderland M. (2011). Psychometric comparison of the phq-9 and BDI-II for measuring response during treatment of depression. Cogn. Behav. Ther. 40, 126–136. 10.1080/16506073.2010.550059 25155813

[B78] van Ballegooijen W. Cuijpers P. van Straten A. Karyotaki E. Andersson G. Smit J.. (2014). Adherence to internet-based and face-to-face cognitive behavioural therapy for depression: a meta-analysis. PLoS ONE 9:e100674. 10.1371/journal.pone.0100674 25029507 PMC4100736

[B79] van Gemert-Pijnen J. E. Kelders S. M. Bohlmeijer E. T. (2014). Understanding the usage of content in a mental health intervention for depression: an analysis of log data. J. Med. Internet Res. 16:e27. 10.2196/jmir.2991 24486914 PMC3936274

[B80] Webb C. A. Forgeard M. Israel E. S. Lovell-Smith N. Beard C. Björgvinsson T. (2022). Personalized prescriptions of therapeutic skills from patient characteristics: an ecological momentary assessment approach. J. Consult. Clin. Psychol. 90, 51–60. 10.1037/ccp0000555 33829818 PMC8497649

[B81] Whiteford H.arvey A. P Degenhardt Louisa P. Rehm Jürgen, P. Baxter A.manda J. M.. (2013). Global burden of disease attributable to mental and substance use disorders: findings from the global burden of disease study 2010. Lancet 382, 1575–1586. 10.1016/S0140-6736(13)61611-6 23993280

[B82] WHO. (2022). Mental Health and COVID-19: Early Evidence of the Pandemic's Impact. World Health Organization: Scientific Brief 2, 1–11.

[B83] Williams R. Farquharson L. Palmer L. Bassett P. Clarke J. Clark D. M.. (2016). Patient preference in psychological treatment and associations with self-reported outcome: national cross-sectional survey in england and wales. BMC Psychiatry 16, 1–8. 10.1186/s12888-015-0702-8 26768890 PMC4714467

[B84] WMA. (2013). World medical association declaration of Helsinki: ethical principles for medical research involving human subjects. JAMA 310, 2191–2194. 10.1001/jama.2013.281053 24141714

[B85] Xu R. (2003). Measuring explained variation in linear mixed effects models. Stat. Med. 22, 3527–3541. 10.1002/sim.1572 14601017

